# Adaptation and psychometric validation of Diabetes Health Profile (DHP-18) in patients with type 2 diabetes in Quito, Ecuador: a cross-sectional study

**DOI:** 10.1186/s12955-021-01818-5

**Published:** 2021-07-31

**Authors:** Ikram Benazizi, Mari Carmen Bernal-Soriano, Yolanda Pardo, Aida Ribera, Andrés Peralta-Chiriboga, Montserrat Ferrer, Alfonso Alonso-Jaquete, Jordi Alonso, Blanca Lumbreras, Lucy Anne Parker

**Affiliations:** 1 Department of Public Health, Universidad Miguel Hernández, Sant Joan d’Alacant, Alicante, Spain; 2 CIBER de Epidemiología y Salud Pública (CIBERESP), Madrid, Spain; 3 Health Services Research Group, IMIM (Hospital del Mar Medical Research Institute), Barcelona, Spain; 4 Universitat Autònoma de Barcelona, Barcelona, Spain; 5 Cardiovascular Epidemiology and Research Unit, University Hospital and Research Institute Vall d’Hebron (VHIR), Barcelona, Spain; 6 Instituto de Salud Pública, Pontificia Universidad Católica del Ecuador, Quito, Ecuador; 7 Unidad Docente de Medicina Preventiva y Salud Pública de Cantabria, Consejería de Sanidad de Cantabria, Santander, Spain; 8 Department of Experimental and Health Sciences, Pompeu Fabra University, Barcelona, Spain

**Keywords:** Diabetes Health Profile-18, Diabetes mellitus, Quality of life, Validity, Reliability, Ecuador

## Abstract

**Introduction:**

The Diabetes Health Profile (DHP‐18), structured in three dimensions (psychological distress (PD), barriers to activity (BA) and disinhibited eating (DE)), assesses the psychological and behavioural burden of living with type 2 diabetes. The objectives were to adapt the DHP‐18 linguistically and culturally for use with patients with type 2 DM in Ecuador, and to evaluate its psychometric properties.

**Methods:**

Participants were recruited using purposive sampling through patient clubs at primary health centres in Quito, Ecuador. The DHP-18 validation consisted in the linguistic validation made by two Ecuadorian doctors and eight patient interviews. And in the psychometric validation, where participants provided clinical and sociodemographic data and responded to the SF-12v2 health survey and the linguistically and culturally adapted version of the DHP-18. The original measurement model was evaluated with confirmatory factor analysis (CFA). Reliability was assessed through internal consistency using Cronbach’s alpha and test–retest reproducibility by administering DHP-18 in a random subgroup of the participants two weeks after (n = 75) using intraclass correlation coefficient (ICC). Convergent validity was assessed by establishing previous hypotheses of the expected correlations with the SF12v2 using Spearman’s coefficient.

**Results:**

Firstly, the DHP-18 was linguistically and culturally adapted. Secondly, in the psychometric validation, we included 146 participants, 58.2% female, the mean age was 56.8 and 31% had diabetes complications. The CFA indicated a good fit to the original three factor model (χ2 (132) = 162.738, p < 0.001; CFI = 0.990; TLI = 0.989; SRMR = 0.086 and RMSEA = 0.040. The BA dimension showed the lowest standardized factorial loads (λ) (ranging from 0.21 to 0.77), while λ ranged from 0.57 to 0.89 and from 0.46 to 0.73, for the PD and DE dimensions respectively. Cronbach’s alphas were 0.81, 0.63 and 0.74 and ICCs 0.70, 0.57 and 0.62 for PD, BA and DE, respectively. Regarding convergent validity, we observed weaker correlations than expected between DHP-18 dimensions and SF-12v2 dimensions (r > −0.40 in two of three hypotheses).

**Conclusions:**

The original three factor model showed good fit to the data. Although reliability parameters were adequate for PD and DE dimensions, the BA presented lower internal consistency and future analysis should verify the applicability and cultural equivalence of some of the items of this dimension to Ecuador.

## Background

Diabetes mellitus (DM) is a high priority public health problem. It is the most frequent chronic disease in the world and, in 2014, affected 422 million people. According to the World Health Organization, people with type 2 Diabetes mellitus (T2DM) represent 90% of all diabetics. The prevalence of T2DM has increased more rapidly in low- and middle-income countries than in high-income countries, as is the case in Latin America and Ecuador [1]. In 2016, the prevalence of T2DM in Ecuador was estimated at 7.3% and has been rising significantly in all age groups [2–5]. According to data from the STEPS Survey of Ecuador in 2018, the prevalence of diabetes was 6.6% in both sexes (6.6% in men and 6.5% in women) of the Ecuadorian population between 18 and 69 years of age, and increased to 10.7% in the age group between 45 and 69 years in both sexes [6].

T2DM is the most common metabolic cause of mortality, due to its complications and associated pathologies [7]. It negatively affects quality of life [8], defined as a person's individual perception of the physical, emotional and social state [9], as a result of associated physical disabilities and mental health problems [10]. Clinical measures can provide a good estimate of disease control, but the ultimate goal of DM care is to maintain or improve the patient's quality of life [11].

There are generic instruments to measure quality of life that can be used both in the general population and in all disease groups [12, 13]. However, specific instruments have been developed to measure specific effects of diseases and are more responsive to changes. Disease-specific instruments can help determine which conditions best explain a patient's limitations in physical and / or mental function, and, therefore, are more useful in outcome research, health care cost studies, and clinical practice [14].

In Ecuador, advanced age, longer disease duration, hypertension and kidney disease are associated with a lower health related quality of life in patients with T2DM [15, 16]. In addition, a direct relationship was found between low socioeconomic status and the development of the disease [17].

Despite the rapid growth in the prevalence of T2DM and the existence of different instruments to measure quality of life in diabetic patients, none of them have been linguistically or psychometrically validated in Ecuador. Although there is a wide description of different questionnaires to assess quality of life in diabetic patients [18], such as the Diabetes Care Profile, which aims at assessment of factors important in a patient's adjustment to diabetes and its treatment in daily life and which consists of 234 items, the Appraisal of Diabetes Scale, which aims at Assessment of diabetes-related distress and which consists of 7 items, the Diabetes Distress Scale, which aims to Measure of diabetes-related emotional distress for use in research and clinical practice and which consists of 17 items, among others [19]. We chose the Diabetes Health Profile (DHP) because of its advantages over other diabetes-specific patient reported outcome measures. It is a specific instrument to evaluate psychological and behavioural impact of living with diabetes [20]. It generates a health profile that measures psychological distress, barriers to activity, and uninhibited eating. Each answer is rated on a scale, and the scores by dimension are presented on a scale in which a higher DHP value is associated with a worse perception of quality of life. The short version of DHP with 18 items has been used in different countries, demonstrating adequate metric properties [21–23].

The objectives of this study are to adapt the Diabetes Health Profile-18 (DHP‐18) both linguistically and culturally for use with patients with T2DM in Ecuador, and to evaluate its psychometric properties.

## Methods

### Participants

We included type 2 diabetic patients, who were at least 18 years of age, had been diagnosed for at least 12 months, resided in Quito with no intention of moving in the near future and were native Spanish speakers. Recruitment to the study used purposive sampling through a patient club for people with diabetes at the Chimbacalle Health Center and contacts from health promoters from several health centres in Quito (Número 1, Jardín del Valle, Cotocollao, Jaime Roldos Aguilera, Corazón de Jesus, Comité del Pueblo, San Antonio de Pichincha, Colinas del Norte, Pomasqui, Carcelén Bajo, El Condado, Mena del Hierro, La Bota, Pisulí, Puellaro, Chavezpamba, Cotocollao Alto and Calacalí).

In this setting, diabetic patient’s clubs are sometimes established in primary health care centres, either by initiative of the health staff or the patient’s themselves. The role of patient clubs is to motivate patients through the exchange of experiences among its members, in addition to the orientation, advice and guidance offered by health professionals on behaviour modification (physical activity/diets) [24, 25].

Our selection sought to include a group of patients that was heterogeneous in terms of sex, age and level of education. All participants gave their consent to participate in the study.

### Procedure

The interviews were carried out between February and July 2020. The DHP-18 validation process consisted of 2 phases.

#### Linguistical and cultural adaptation

Two Ecuadorian medical researchers reviewed the original version of the DHP-18 (English) and the existing translation (Spanish for the United States) to assess the cultural and linguistic relevance for its use in Ecuador. They suggested some changes in text, as well as the reasons for these changes and provided a new recommended translation. Changes were discussed with the other members of the team and a new adapted version of the questionnaire was proposed. Subsequently, 2 different researchers carried out interviews to assess the linguistic and cultural understanding of the adapted questionnaire with 8 people with T2DM of Ecuadorian nationality in the Chimbacalle Health Centre. Participants were asked to answer the questions and then, the necessary time was recorded, the answer options were discussed, the wording that was difficult to understand was commented, and alternative wording was suggested based on the participants’ own words. A second adapted version was proposed. The interviews were recorded and transcribed verbatim for analysis. Finally, participants' responses were summarized in a pilot test report including recommended changes and suggestions. The report was then sent to the original authors of the questionnaire for verification and approval.

#### Psychometric validation

Firstly, we recruited 146 participants for the baseline test where they responded to the questions posed in the tool previously linguistically validated DHP-18 instrument in Ecuador and in another tool (SF-12v2 in its version for use in Ecuador) [26] in order to assess the correlation with generic quality of life as a construct validity test. Two weeks later, we assessed the intra-observer reliability of the new tool in a random sample of 75 of the previously interviewed patients, where only DHP-18 was retested, along with the following question: “Compared to the last time you completed the questionnaire, how do you assess your condition today? (1) unchanged, (2) improved, (3) greatly improved, (4) impaired or (5) highly impaired”.

### Data collection

The 8 interviews carried out during the linguistic and cultural adaptation were held face to face but given the situation generated by the COVID19 pandemic [27], the data for the psychometric validation was collected through individual telephone interviews. Responses were digitally recorded by the interviewer using the Kobo toolbox (http://www.kobotoolbox.org/) free open-source software on electronic tablets. Informed consents were provided orally and were audio recorded.

#### DHP-18 questionnaire

Participants responded to the adapted version of DHP-18. We used the Diabetic Health Profile (DHP) -18 because it is a shortened version of DHP-1, a specific instrument for measuring the psychological and behavioural impact of type 1 diabetes. We decided to use the short version of the DHP because it can be used in people with both type 1 and type 2 diabetes aged 11 and older. And because the instrument has demonstrated adequate metric properties and its completion time is approximately 5–6 min. Items are scored using a 4-point Likert-type scale ranging from 0 to 3. Items are provided with one of four sets of responses (1) never, sometimes, generally, always; (2) never, sometimes, often, very often; (3) not at all, a little, a lot, very much; and (4) very likely, quite likely, unlikely, not at all likely. The raw subscale scores are transformed into a common score range from 0 to 100, with 0 representing no dysfunction.

The DHP-18 consists of three dimensions: psychological distress (includes questions like depressed from diabetes; more arguments or upsets at home than there would be if you did not have diabetes; losing your temper over unimportant things; etc.), barriers to activity (includes questions like food controls life; difficult staying out late; avoid going out when sugar is low; etc.) and disinhibited eating (includes questions hard to say no to food you like; ease of stopping when you eat; wish there were not so many nice things to eat; etc.).

#### SF-12 v2

The SF-12 v2 is an instrument for measuring health-related quality of life [26], based on SF-36. It includes twelve items, has an application time of approximately two minutes, and is used to evaluate the degree of well-being and functional capacity of people over 14 years of age. The response options form Likert-type scales (where the number of options varies from three to six points, depending on the item), which assess intensity and / or frequency of people's health status. The score ranges from 0 to 100, where the higher score implies a better health-related quality of life. The SF-12v2 has demonstrated adequate validity and reliability in the United States and internationally, and the Spanish version has been used successfully in Latin America and with Spanish-speaking populations in the United States. Investigations that use these twelve items of the SF have verified that the instrument is a valid and reliable measure in Latin American countries such as Colombia and Chile in adult population, and a translated version is available for Ecuador.

The SF12v2 includes questions related to health status and limitations in doing activities, problems with work or other regular daily activities due to physical health, due to emotional problems, pain, feelings, etc.

#### Sociodemographic and clinical variables

We collected sociodemographic and clinical variables (all self-reported by the participants): age, sex, marital status, ethnicity (mestizo or other minorities. The mestizos are an ethnicity composed of Spanish and indigenous heritages), educational level, monthly income, employment status, smoking status, alcohol intake, weight, height, duration of illness, use of medications, diabetes complications and comorbidities.

### Statistical analysis

We included descriptive statistics through frequencies, the mean (standard deviation) or the median (interquartile range), as appropriate. The psychometric characteristics of the DHP-18 were assessed according to consensus-based standards for the selection of health status measurement instruments (COSMIN) guidelines [28]. Missing values for the DHP-18 and SF-12 v2 were substituted with the mean of the completed questions for those dimensions in which ≥ 50% of questions had been completed [29, 30].

We evaluated floor and ceiling effects by calculating the percentage of patients scoring either the lowest or highest possible dimensional scores. If more than 15% of respondents achieve the lowest or highest possible score, then floor or ceiling effects are present [31].

Statistical analyses were performed using Stata Version 15 (StataCorp LP; College Station, TX) and R software, version R 4.0.0 (R Core Team. R: A language and environment for statistical computing. R Foundation for Statistical Computing, Vienna, Austria; http://www.R-project.org) was used to perform the confirmatory factor analysis. The level of statistical significance was set at p < 0.05.

#### Structural validity

We performed a confirmatory factor analysis (CFA) because the factor structure had already been determined [32] and confirmed for other language translations [23]. In this case, we used CFA using Diagonally Weighted Least Squares (DWLS) [33–35] to test the hypothesis that the general construct of DHP is composed of three individual and correlated factors: psychological distress (6 items), activity barriers (7 items) and disinhibited eating (5 items). To estimate the model fit, we used the following criteria: Values > 0.95 for the Tucker-Lewis index (TLI) or for the comparative fit index (CFI) and the root mean square error of approximation (RMSEA) < 0.06 or the standardized root mean square residual (SRMR) < 0.08 are considered as a good model fit [36, 37]. The magnitudes of factor loadings of 0.3 or greater were considered suitable.

#### Reliability

To measure internal consistency reliability, we used Cronbach’s alpha coefficient, where values > 0.7 are considered as acceptable [36]. The homogeneity of items was verified by the analysis of item-rest and inter-item correlations for the items constituting each dimension of the scale. The usual rule of thumb is that an item should correlate between 0.3 and 0.7 with the total score of the factor (excluding that item), using Pearson’s coefficient. Additionally, average inter-item correlations for items in the same factor should correlate moderately, between 0.15 and 0.5, to ensure that they measure the same construct but not so closely as to be too redundant [38].

We measured test–retest reliability in patients reporting no-change in the global assessment of change question. To measure test–retest reliability we considered that the individual’s health was significantly better if they responded, “much better” or “somewhat better” in the global assessment, or significantly worse if they responded “somewhat worse” or “much worse” [39]. We used the intraclass correlation coefficient (ICC) under a 2-way random effects model with absolute agreement [40], and its associated 95% confidence interval. We considered that a questionnaire exhibits substantial reliability when ICC is between 0.40 and 0.75, and greater than 0.90 represents excellent reliability [36].

Measurement errors were determined by calculating the standard error of measurement (SEM) and the smallest detectable change (SDC). We calculated SEM by the square root of the error variance derived from analysis of variance (ANOVA), two-way ANOVA with repeated measures [41]. The SDC_individual_ and SDC_group_ was calculated with the following formulas (41):

$${\text{SDC}}_{{{\text{individual}}}} = { 1}.{96 }* \, \surd {2 }*{\text{ SEM}}$$

SDC_group_ = (SDC_individual_ /√n); n: number of subjects in the sample.

We estimated the minimally important difference (MID) for each DHP-18 dimension using three distribution-based methods to estimate MID: 0.2 and 0.5 standard deviation (SD) and SEM estimations. Formulas:

$$\begin{aligned} & 0.{\text{2SD}} = \, 0.{2}*{\text{ SD}}_{{{\text{basaline}}}} \\ & 0.{\text{5SD}} = \, 0.{5}*{\text{ SD}}_{{{\text{basaline}}}} \\ & {\text{1SEM}} = {\text{SEM}} \\ \end{aligned}$$

We also estimated Cohen’s d effect size (ES) of the change in DHP-18 dimensions for those reporting a small but important change and those reporting no changes in global assessment rating. Cohen’s d was calculated with the following formula (42):

$${\text{ES }} = \, \left( {{\text{Score}}_{{{\text{baseline}}}} - {\text{Score}}_{{{\text{retest}}}} } \right)/{\text{SD}}_{{{\text{basaline}}}}$$

SD_basaline_: Standard deviation of baseline score.

An effect size of 0.2 was considered small, 0.5 moderate and 0.8 large [43].

#### Construct validity

We assessed construct validity of the DHP questionnaire using three approaches. Firstly, we assessed convergent validity using binary correlation analysis (Spearman’s r- due to non-normal value distributions) of the DHP-18 and SF-12v2. Before starting the analysis, we set up the following a priori hypothesis: (1) Scores of “psychological distress” dimension in DHP-18 correlate negatively with scores of “mental health” dimension in SF-12v2. (2) Scores of “activity barriers” dimension in DHP-18 correlates negatively with “physical dimension” in SF-12v2. (3) Scores of “disinhibited eating” dimension in DHP-18 correlates negatively with “physical dimension” in SF-12v2.

Secondly, we explored discriminant validity by comparing the correlation among the three dimensions of the DHP-18 scale.

Thirdly, we evaluated known-group validity by comparing DHP-18 scores in patients according to sex, education level, obesity, and clinical characteristics such as duration of diabetes, presence of comorbidities and/or diabetes-related complications using a Student’s t-test or ANOVA. We tested the following pre-defined hypotheses:

**H1:** Individuals with longer duration of illness would have higher DHP-18 scores (poorer quality of life) than those with shorter illness duration [44].

**H2:** Obese individuals would have higher DHP-18 values (poorer quality of life) than non-obese individuals [45].

**H3:** Women would report higher DHP-18 values (poorer quality of life) than men [46].

**H4:** Individuals with comorbiditie**s** would have higher DHP-18 values (poorer quality of life) [44].

**H5:** Individuals with a higher education level would have lower DHP-18 values (better quality of life) than those with a lower education level [47, 48].

**H6:** Individuals with diabetes-related complications would have higher DHP-18 values (poorer quality of life) than patients without complications [44].

## Results

### Linguistic and cultural adaptation

Two Ecuadorian medical researchers modified some linguistic expressions in the Spanish version for the United States in items 2, 3, 4, 5, 6, 10, 11, 12, 13, 14, 15 and 18 and in some answer options. In the linguistic and cultural review, six women and two men participated: a 28-year-old person, a 49-year-old person and a 52-year-old person, and the rest of the participants were over 70 years old. They made further changes to items 5, 6, 10 and 12 and proposed reformulation of some expressions. Most modifications were minor linguistical issues to use terms more commonly used in Ecuador, for example the expression “staying out” was changed by “going out of the house”, the term “edgy” was changed by “nervous”, and the term "lose your temper" was changed by "get angry easily". Other changes were made to improve comprehension by simplifying technical terms, for example “influenza” was changed to “flu”, “depressed” was changed to “sad”. One of the items was flagged as having potential difficulties because participants would be asked to reflect on their sugar levels, and there was a very low availability of glucometers in homes. The expression "on the low side" was changed to "having low or very low sugar levels". Similarly, in item 6, the word "monitor" was replaced by the expression "take the sugar test" to improve its understanding. The original author approved the new tool, linguistically and culturally adapted to the context of Quito, Ecuador.

### Psychometric validation

We recruited 146 patients diagnosed with T2DM. Table 1 describes the characteristics of the study population. The mean age of the participants was 56.8 years, 58.2% were women and 80.1% were mestizo. The population studied had relatively low educational qualifications, with 56.8% having primary or no education, 27.6% were not working and 61.4% had incomes of less than $375 per month.

**Table 1:** Description of study population

Characteristics	Total n: 146 (%)	Re-test n:75 (%)	p valor
*Age*			
Mean (SD)	56.8 (11.3)	57.5 (10.3)	0.467
*Sex*			
Female	85 (58.2)	41 (54.7)	0.404
Male	61 (41.8)	34 (45.3)	
*Ethnicity*			
Mestizo	117 (80.1)	59 (78.7)	0.683
Other minorities	29 (19.9)	16 (21.3)	
*Civil status*			
Married/cohabitant	99 (67.8)	55 (73.4)	0.127
Separated/divorced	18 (12.3)	10 (13.3)	
Single/widow-widower	29 (19.9)	10 (13.3)	
*Educational level*			
No formal schooling	10 (6.8)	3 (4.0)	0.561
Basic education	73 (50.0)	38 (50.7)	
High school and non-university higher education	47 (32.2)	25 (33.3)	
University studies	16 (11.0)	9 (12.0)	
*BMI*			
18.5–24.9 kg/m^2^	28 (23.3)	12 (18.7)	0.159
25.0–29.9 kg/m^2^	45 (37.5)	22 (34.4)	
≥ 30 kg/m^2^	47 (39.2)	30 (46.9)	
Not reported	26	11	
*Time since diagnosis with diabetes*			
< = 5 years	54 (37.0)	28 (37.3)	0.245
6–10 years	45 (30.8)	27 (36.0)	
> 10 years	47 (32.2)	20 (26.7)	
*Diabetes complications*			
At least one	31 (21.2)	16 (21.3)	0.976
Macrovascular complication*	5 (3.4)	3 (4.0)	0.694
Microvascular complication	28 (19.2)	14 (18.7)	0.872
Ocular	23 (15.8)	11 (7.5)	0.965
Renal	7 (4.8)	3 (2.1)	0.802
Neuropathy	2 (1.4)	0	
Diabetic foot	2 (1.4)	1 (0.7)	0.941
*Comorbidities*			
At least one	84 (57.5)	44 (58.7)	0.776
Hypertension	65 (44.5)	37 (49.3)	0.229
Depression	7 (4.8)	2 (2.7)	0.266
Dyslipidemia	8 (5.5)	4 (5.3)	0.879
Other comorbilidities	10 (6.8)	2 (2.7)	0.051

*NA* Not available

*Includes: Ischemic Heart Disease, Cerebrovascular Disease, Heart Failure

Regarding diabetes medication, the majority were on oral antidiabetic therapy (66.2%), 11.7% of patients were treated with insulin, 2.1% with only diet and the rest (20%) were on combined therapy (oral + insulin). We found that 37.5% were overweight and 29.5% were obese.

Of the 146 respondents, 135 (92.5%) answered the 18 items of DHP-18; 9 (6.2%) omitted one item and 2 (1.3%) omitted two or more items. The missing values were in items 4, 5, 7,12, 13 and 14. One item (question 14) of the DHP-18 version showed unbalanced responses with 75% of respondents reporting never.

Seventy-five (51.4%) participants were retested for DHP-18. There were no differences in socio-demographic or clinical characteristics between participants who were retested and those who were not (Table 1). In the DHP-18 retest, there were two missing values in item 4 and two in item 14, the distribution of the missing were 2 participants who did not answer one item and 1 participant did not answer two items.

#### Structural validity

**Fig. 1:**
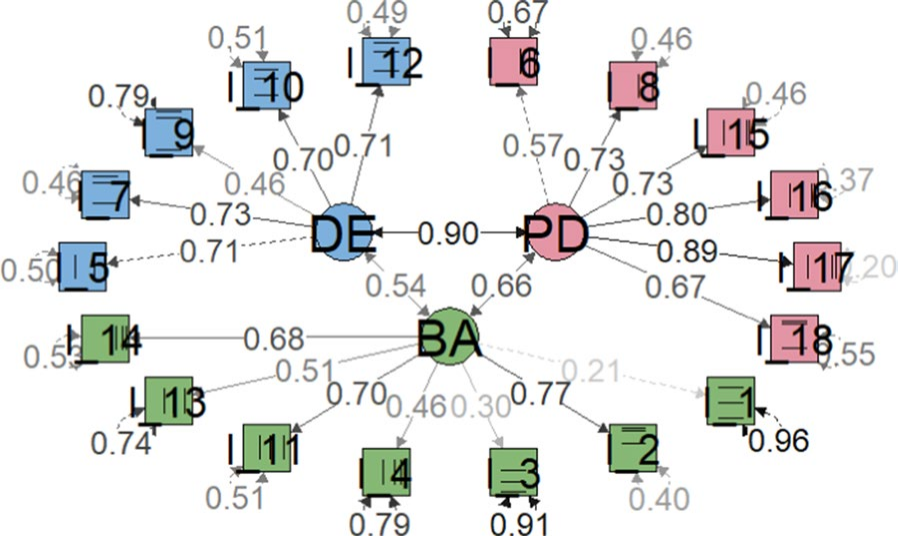
Confirmatory Factor Analysis performed based on the polychoric correlation matrix, using diagonally weighted least squares (DWLS). CFI = 0.990. TLI = 0.989. RMSEA = 0.040 (90% confidence interval: 0.011–0.059). SRMR = 0.086

CFA with values of χ^2^ (132) = 162.738, p < 0.001; CFI = 0.990; TLI = 0.989; SRMR = 0.086 and RMSEA = 0.040 indicated a good fit to the data, except for SRMR. The standardized factorial loads (λ) from each item on their respective factors were all statistically significant (p < 0.001) and ranged from 0.57 to 0.89, from 0.21 to 0.77 and from 0.46 to 0.73 for psychological distress, barriers to activity and disinhibited eating, respectively. The covariance between the three latent variables ranged from 0.54 to 0.90, with psychological distress and disinhibited eating presenting the highest covariance. And two items (questions 1 and 3) showed a λ value below 0.3, using a one-factor model (Fig. 1). When we repeated the analysis excluding these 2 items, we observed a significant improvement in all the indicators, including the SRMR, which was the only one that showed a value slightly higher than recommended (CFI = 0.997; TLI = 0.996; RMSEA = 0.027 (90% confidence interval: 0.000–0.052); SRMR = 0.078).

#### Reliability

Overall Cronbach’s alpha was 0.77 and dimensional alphas were 0.81, 0.63 and 0.74 for psychological distress, barriers to activity and disinhibited eating, respectively. The three dimensions were in a suitable range (0.15–0.50) for average interitem correlation values which ranged from 0.38 to 0.47, from 0.17 to 0.26 and from 0.32 to 0.41 for psychological distress, barriers to activity and disinhibited eating, respectively. Item-rest correlation values ranged from 0.39 to 0.61 for disinhibited eating while values ranged from 0.43 to 0.72 for psychological distress dimension, where item 17 showed the highest value (0.72) and ranged from 0.07 to 0.47 for barriers to activity, where item 1 showed the lowest value (0.07).

When we repeated the analysis excluding question 1 which had an item-rest correlation value below 0.30 (value: 0.07) and a λ value < 0.3 in the barriers to activity dimension, the dimensional and overall Cronbach’s alpha changed to 0.67 and 0.76, respectively. When question 17 (item-rest correlation value slightly higher than 0.7) was excluded from psychological distress dimension, the dimensional and overall Cronbach’s alpha changed to 0.75 and 0.76, respectively.

ICC values for a total of 75 retested participants (Table 2) were 0.70 (95%CI: 0.57, 0.80), 0.67 (95%CI 0.56–0.77), 0.73 (95%CI 0.64–0.81) for psychological distress, barriers to activity and disinhibited eating, respectively.

**Table 2:** Test–retest reliability of the Diabetes Health Profile-18 subdimensions overall, and considering a reported change in global assessment of health

Global assessment of health	N	Baseline, mean (sd)	Retest, mean (sd)	Mean differences (sd)	ES	Minimally important difference			SDC_ind_	SDC_gru_	ICC (95%CI)
						0.2SD	0.5SD	1SEM			
*PD*											
Stayed the same	39	22.4 (16.3)	21.5 (13.7)	0.8 (11.9)	0.05	3.27	8.17	8.43	23.37	2.65	0.69 (0.48–0.83)
Worse	15	28.9 (17.7)	26.3 (17.5)	2.6 (10.9)	0.14	3.54	8.86	7.70	21.33	3.89	0.81 (0.53–0.93)
Better	21	33.9 (23.2)	31.7 (24.8)	2.1 (20.6)	0.09	4.65	11.61	14.56	40.37	6.23	0.64 (0.30–0.84)
Total	75	26.9 (19.2)	25.3 (18.5)	1.5 (14.5)	0.08	3.84	9.59	10.26	28.45	2.32	0.70 (0.57–0.80)
*BA*											
Stayed the same	39	35.2 (15.7)	37.8 (20.4)	− 2.6 (17.6)	0.17	3.14	7.84	10.01	27.75	2.59	0.66 (0.50–0.79)
Worse	15	49.3 (13.2)	56.7 (19.2)	− 7.4 (19.3)	0.56	2.63	6.58	11.38	31.55	4.81	0.22 (− 0.06,0.58)
Better	21	40.1 (20.1)	43.5 (24.3)	− 3.4 (20.1)	0.17	4.01	10.03	11.60	32.16	4.05	0.71 (0.51,0.86)
Total	75	39.4 (17.2)	43.2 (22.3)	− 3.8 (18.3)	0.22	3.45	8.61	12.67	29.57	1.99	0.67 (0.56–0.77)
*DE*											
Stayed the same	39	30.0 (19.0)	32.1 (21.1)	− 2.1 (19.9)	0.11	3.80	9.51	11.70	32.42	3.04	0.66 (0.50–0.80)
Worse	15	41.8 (18.6)	37.3 (20.4)	4.4 (13.9)	0.24	3.72	9.3	8.06	22.33	3.33	0.82 (0.64–0.93)
Better	21	40.3 (20.4)	39.4 (25.1)	0.9 (18.6)	0.05	4.08	10.19	10.76	29.85	3.76	0.77 (0.59–0.89)
Total	75	35.3 (19.8)	35.2 (22.1)	0.1 (18.5)	0.003	3.97	9.92	10.74	29.78	2.00	0.73 (0.64–0.81)

*PD* Psychological distress, *BA* Barriers to activity, *DE* Disinhibited eating, *ES* effect size, *SD* standard deviation, *SEM* standard error of measurement, *SDC* small detectable change, *ICC* intraclass correlation coefficient

Among the retest participants, thirty-nine (52%) reported that their condition was unchanged from baseline to retest (ICC values in Table 2) and 36 (48%) reported that their condition had changed from baseline to retest. Fifteen (20%) participants reported that their condition had improved and 21 (28%) reported that their condition had deteriorated**.** ICC values for participants reporting that their condition stayed the same were 0.69 (95%CI 0.48–0.83), 0.66 (95%CI 0.50–0.79), 0.66 (95%CI 0.50–0.80) for psychological distress, barriers to activity and disinhibited eating, respectively.

#### Construct validity

Our assessment of convergent validity showed an inverse relationship between DHP-18 dimensions and SF12v2 dimensions and the results verified two of three a priori hypotheses with correlation values between 0.4 and 0.7 (Table 3).

**Table 3:** Convergent validity: correlation (Spearman’s r) between the Diabetes Health Profile-18 dimensions and the SF12v2 dimensions

SF-12v2 dimensions	Diabetes Health Profile (DHP-18)		
	Psychological distress	Barriers to activity	Disinhibited eating
Physical functioning	− 0.268	− 0.316	− 0.322
Role-physical	− 0.021	− **0.435**	− 0.284
Bodily pain	− 0.246	− 0.311	− 0.268
General health	− 0.380	− 0.240	− 0.326
Vitality	− 0.334	− 0.248	− 0.263
Social functioning	− 0.248	− 0.261	− 0.236
Role-emotional	− 0.353	− **0.407**	− **0.412**
Mental health	− **0.517**	− 0.292	− 0.322

All values were significant with p < 0.05; Values > 0.40 in bold

For discriminant validity, correlations between the DHP-18 dimensions were 0.4 or more, ranging from 0.40 to 0.74. The highest correlation was between psychological distress and disinhibited eating (r = 0.74), followed by the correlation between psychological distress and barriers to activity (r = 0.45) and the lowest was the correlation between barriers to activity and disinhibited eating (r = 0.40).

With regard to known-group validity, our results showed the expected tendency in three (H2, H3 and H6) of the 6 initial hypotheses. Compared to individuals with BMI < 30 kg/m^2^, those with BMI ≥ 30 kg/m^2^ (H2) showed higher values for each dimension, although only those associated to disinhibited eating were statistically significant. For H3 and H6, the expected tendency of scores for each dimension was obtained with higher scores in women than men and in patients with diabetes-related complications than those without, but there were no statistically significant differences (Table 4).

**Table 4:** Known group validity of the DHP-18

Hypothesis	Category	n (%)	Psychological distress		Barriers to activity		Disinhibited eating		Global	
			Mean (sd)	p-value	Mean (sd)	p-value	Mean (sd)	p-value	Mean (sd)	p-value
H1	*Duration of disease*			0.13		0.94		0.31		0.33
	≤ 5 years	54 (37.0)	31.17 (25.06)		39.74 (22.05)		37.61 (22.89)		36.29 (20.11)	
	6–10 years	45 (30.8)	26.42 (20.86)		38.75 (14.84)		37.19 (20.46)		34.20 (14.85)	
	> 10 years	47 (32.2)	22.81 (14.75)		38.45 (18.29)		31.70 (19.28)		31.36 (13.45)	
H2	*BMI*			0.12		0.46		**0.01**		0.09
	< 30 kg/m^2^	73 (60.8)	25.19 (18.91)		38.59 (18.78)		31.85 (20.46)		32.25 (15.92.)	
	≥ 30 kg/m^2^	47 (39.2)	29.79 (24.06)		38.94 (18.66)		40.63 (21.58)		36.36. (17.60)	
H3	*Sex*			0.08		**0.04**		0.06		**0.03**
	Female	85 (58.2)	29.08 (20.89)		41.30 (18.17)		37.92 (21.34)		36.29 (15.99)	
	Male	61 (41.8)	24.13 (21.10)		35.83 (30.92)		32.31 (20.39)		30.95 (17.13)	
H4	*Comorbidities*			0.80		0.71		0.45		0.71
	No	62 (42.5)	28.76 (24.01)		40.04 (19.52)		35.34 (23.08)		34.97 (18.95)	
	Yes	84 (57.5)	25.73 (18.61)		38.27 (18.21)		35.75 (19.58)		33.39 (14.76)	
H5	*Educational level*			0.85				0.75		0.89
	Basic education or below	83 (56.9)	28.58 (21.97)		40.81 (17.93)	0.91	36.63 (20.98)		35.57 (31.86)	
	Higher education	63 (43.1)	24.96 (19.75)		36.66 (19.64.)		34.20 (21.26)		32.07 (16.06)	
H6	*Diabetes complications*			0.29		**0.03**		0.10		0.07
	No	115 (78.8)	26.52 (20.86)		37.49 (18.56)		34.44 (21.40)		32.99 (16.49)	
	Yes	31 (21.2)	28.85 (21.96)		44.67 (18.57)		39.78 (19.49)		38.04 (16.79)	

Regarding hypotheses H1, H4 and H5, score patterns were different from those expected. Individuals with longer duration of illness (H1) had lower scores reflecting improved quality of life, although the differences were not statistically significant. Similarly, regarding educational level (H5), scores did not show a clear tendency, with the exception of lower scores for barriers to activity dimension with increasing educational level. Finally, there were no differences by presence of comorbidities (H4) but we found differences between patients with or without specific comorbidities such as hypertension and depression. Having hypertension was associated with better evaluation of two dimensions (psychological distress and disinhibited eating), while depression was associated with worse evaluation of two dimensions (barriers to activity and disinhibited eating) (Table 4).

## Discussion

In the present study, we linguistically and culturally adapted the DHP-18 and investigated its psychometric properties in people resident in Quito, Ecuador. Satisfactory psychometric properties were observed in a substantial number of aspects. The factor structure was adequate but with 2 items, belonging to the dimension of barriers to activity, which were loaded below the recommended value. Although reliability parameters were adequate for psychological distress and disinhibited eating dimensions, the barriers to activity presented lower internal consistency and future analysis should verify the applicability and cultural equivalence of some of the items of this dimension to Ecuador.

Except for the dimension of barriers to activity, a good internal consistency was found. The internal consistency of the dimension of barriers to activity contrasts with another study [21] and may be related to the different populations of patients investigated [21, 22, 32], since in some studies of people with both type 1 or 2 diabetes were included. Based on a more detailed analysis of the total item statistics, we observed that the elimination of items 1 and 17, with the lowest and highest item-rest correlation values, did not produce significant increases in overall and dimensional consistency, as observed in another study [23].

The test–retest reliability showed substantial reliability values in accordance with the recommendations of the literature [36]. And the sample size used is within the recommended ranges in psychometric validation studies, which could be considered a strength of our study [49].

Regarding convergent validity, a strong correlation was shown between the dimension of psychological distress of the DHP-18 and the mental health dimension of the SF-12v2 and between the dimension of barriers to activity of the DHP-18 and the role physical dimension of the SF-12v2, corroborating two of the predefined hypotheses. Similar results have been observed in previous studies [21, 23]. However, the uninhibited eating dimension was related to the emotional role dimension and not to the physical role dimension as was hypothesized based on other studies [21].

Discriminant validity showed adequate correlations between the 3 dimensions, higher than those indicated in the literature. These results differ from other studies that showed an overall low correlation between the dimensions of DHP-18 [20, 23].

Regarding the known group validity, our results showed the expected trend in three of the 6 initial hypotheses. Regarding the hypothesis related to educational level, lower scores were given for the dimension barriers to activity with the increase in educational level. Regarding comorbidities there were also significant differences for specific comorbidities such as hypertension and depression. These results are corroborated with other studies where it was seen that the presence of hypertension resulted in a significantly lower score in the disinhibited eating dimension [50]. In the case of the duration of the disease, and despite the fact that the differences were not significant, we did see that people with a disease of longer duration reported a better quality of life. One possible explanation is that the longer the disease lasts, the more likely the patient is able to adapt to the care requirements including behaviour modification [51, 52].

The CFA indicated an adequate fit to the original three-factor model, with the exception of the SRMR indicator. The dimension barriers to activity showed the lowest standardized factorial loads, while for the dimensions psychological distress and uninhibited eating, they were adequate. Using a one-factor model, two of the 18 items, both of the activity barriers dimension, were loaded below the recommended value of 0.3, item 3 loaded with a value of 0.3 and item 1 with a value less than 0.3. Regarding item 1, the problem may be due to a lack of understanding, perhaps not at the linguistic level, but of concepts, since in the linguistic comprehension and cultural adaptation interviews, it was observed that the question was simple, short and easily understood, but sometimes people were unsure whether food “controlling one’s life” referred to the need to observe and take care of one’s diet, or whether it referred to having your life structured and organized around food, such as the timing of meals, the physical exercise depending on the amount of food, etc. Perhaps a clarification with examples could be added to overcome this issue in future uses of the questionnaire in Ecuador. Item 3, about “tied to meal times” was also flagged as potentially problematic in the initial round of reviews by the 2 medical researchers, who evaluated linguistic understanding and cultural adaptation. It should be added that we carried out a new confirmatory factor analysis, eliminating these 2 items and observed a significant improvement in all the indicators, including the SRMR, which was the only one that showed a value slightly higher than recommended.

This study has some limitations in addition to the factors discussed above. Although the DHP-18 can be used with people with either Type 1 or Type 2 diabetes, the psychometric test was not performed in type 1 diabetes, limiting the applicability of the results to those patients with type 2 diabetes. In addition, an important factor to take into account is the context in which the study was carried out, in a pandemic situation it is difficult to assess the possible changes produced and there may be factors external to the disease that can influence the results, especially of repeatability-concordance, due to changes in the context produced quickly and that can affect the quality of life of patients. Another limitation is that SF12v2 summary scores for physical and mental health can be misleading if proprietary scores are used, as a low physical health summary score tends to inflate the mental health summary score and vice versa. So this must be taken into account when interpreting the results [53, 54]. Despite this, the results are significant and similar to those obtained in other studies.

## Conclusions

The strength of this study lies in the fact that this is the first adaptation and validation of a questionnaire to assess the quality of life in diabetic patients in Ecuador. Hence, it provides a practical tool to evaluate aspects such as self-control of food intake, limitations, barriers and anxiety related to daily activities, feelings, emotions, mood and irritability in people with diabetes.

The study adds to the evidence for DHP-18, showing that it is a short, acceptable, valid and reliable instrument to measure the impact of living with diabetes from a patient perspective. However, future analysis should verify the applicability and cultural equivalence of some of the items of barriers to activity dimension to Ecuador. Using DHP-18 enables clinicians to conduct an appropriate educational or therapeutic intervention to alleviate or address dysfunctional life outcomes for people living with diabetes.

## Data Availability

The datasets analysed during the current study are available from the corresponding author on reasonable request.

## Abbreviations

PD: Psychological distress
BA: Barriers to activity
DE: Disinhibited eating
DM: Diabetes mellitus
T2DM: Type 2 Diabetes mellitus
DHP: Diabetes Health Profile
DHP‐18: Diabetes Health Profile-18
CFA: Confirmatory factor analysis
DWLS: Diagonally Weighted Least Squares
TLI: Tucker-Lewis index
CFI: Comparative fit index
RMSEA: Root mean square error of approximation
SRMR: Standardized root mean square residual
ICC: Intraclass correlation coefficient
SEM: Standard error of measurement
SDC: Smallest detectable change
MID: Minimally important difference
SD: Standard deviation
ES: Effect size
